# An Account of the Practice of One of the Physicians of the Westminster General Dispensary, and of the Western Dispensary, from the 20th of May, to the 20th of June

**Published:** 1806-07

**Authors:** Samuel Fothergill

**Affiliations:** Southampton Street, Strand


### *An Account of the Practice of one of the Physicians of the Westminster General Dispensary, and of the Western Dispensary, from the 20th of May, to the 20th of June.*

| | | | |
|---|---|---|---|
| ACUTE DISEASES. | | Scrophula - - | 2 |
| Peripneumony - - | 2 | Epilepsy - - - | 2 |
| Pleurisy - - - | 2 | Chronic Rheumatism | 13 |
| Acute Rheumatism - | 6 | Lumbago and Sciatica | 4 |
| Inflammatory Sore-Throat | 3 | Pleurodyne - - | 3 |
| Synochus - - - | 2 | Gastrodynia - - | 9 |
| Catarrh - - - | 1 | Enterodynia - - | 4 |
| Hæmoptoe - - | 3 | Dyspepsia - - | 9 |
| Ophthalmia - - | 1 | Scirrhous Liver - | 1 |
| Urticaria - - | 3 | Hypochondriasis - | 2 |
| Cholera Morbus - | 1 | Cephalalgia and Vertigo | 7 |
| Measles - - - | 1 | Cramp - - - | 1 |
| Hooping Cough - | 1 | Palpitatio - - - | 2 |
| CHRONIC DISEASES. | | Paralysis - - - | 3 |
| Asthenia - - | 13 | Diarrhœa - - - | 6 |
| Cough and Dyspnœa | 12 | Intestinal Hæmorrhage | 1 |
| Peripneumonia Notha | 1 | Constipation & Vomiting | 3 |
| Pulmonary Consumption | 6 | Worms - - - | 4 |

Ascites

| | | | |
|---|---|---|---|
| Ascites and Anasarca | 6 | Porrigo - - - | 3 |
| Gravel and Dysuria - | 4 | DISEASES OF WOMEN. | |
| Gleet - - - | 1 | Chlorosis - - - - | 2 |
| Syphilis - - - | 1 | Amenorrhœa - - | 6 |
| Carbuncle - - - | 1 | Menorrhagia - - | 4 |
| Herpes - - - | 1 | Dysmenorrhœa - - | 1 |
| Lepra Alphos - - | 1 | Abortus - - - - | 2 |
| Prurigo - - - | 3 | Uterine Hæmorrhagy | 1 |
| Prurigo Senilis - - | 1 | Schirrus Uteri - - | 1 |
| Psora* - - - | 4 | Prolapsus Uteri - | 1 |
| Psoryasis Diffusa - | 1 | Leucorrhœa - - | 3 |
| Psoryasis Infantilis - | 1 | Diseases of Infants - | 9 |

The weather during the last month has been very dry and warm; on some days, indeed, the heat was rather oppressive. The diseases within this period, though numerous, have generally been mild; none of the acute complaints which occurred were very urgent. The cases of acute rheumatism were particularly slight, and chiefly originated from exposure to a stream of air whilst in a state of perspiration.

Workmen and labourers, after working hard, are apt to sit down in the open air and drink their beer, without their coats or waistcoats, and with their shirts open: by this means the perspiration is checked, and the surface of the body too suddenly cooled. By enforcing the danger of such a practice to this useful class of society, we may have the satisfaction of preventing many families the deprivation of their principal means of support, heads of manufactories the loss of able workmen, and the individual himself the pain and anxiety of a tedious illness.

Catarrhal fever is now scarcely to be met with; and the cases of cough and dispnœa have suffered a material diminution since the last Report; many of those now recorded are of long standing In a variable climate, very favourable to the production of pulmonary complaints, we are not to wonder that some people are affected by them at all seasons of the year. The higher classes of society are far from being exempt from complaints which should seem more particularly to affect those only who are exposed to the vicissitudes of temperature, poverty of diet, and hard labour. Crowded rooms artificially heated, bad air, late hours, and quitting a warm suite of apartments after the exercise of dancing, at a time when the external air

---

* See Dr. Willan on Cutaneous Diseases.

air is often cold, are highly injurious to the human frame; and thus the young, the delicate, and the beautiful of the fair sex, who before their constitution has attained the maturity of strength, are exposed to these exciting causes of disease, often become the victims of cough, rheumatism, and consumption.

The influence of the mind upon the animal functions is generally acknowledged, and this influence seems to be greater or less, as the passions are more or less violent. Men of a cold temperament, of a slow, phlegmatic habit, devoid of passion, in whom no wild pulse riots, know little of this influence; and equally free from it is a very different description of men, who by long study and sound philosophy, have assumed a command over their passions, and acquired a serenity of mind which nothing can interrupt or disturb: having calculated the good and the evil of life, they are not easily taken by surprize; and in sickness as in health, retain their self-possession, free from the excitement of hope or the depression of fear. I was disposed to introduce the above observations from the consideration of a case which I have often had occasion to treat. The subject of it is a lady, whose husband, from speculating in business, is liable to vicissitudes of fortune: when his affairs are prosperous, his wife, a beautiful, accomplished, and sensible woman, is full of gaiety and cheerfulness, grows lusty, and enjoys an excellent state of health; a reverse of fortune takes place, when (at whatever season of the year) she is affected with a severe cough and copious expectoration of viscid mucus; her nights are sleepless, her appetite fails, she becomes emaciated, and is subject to pain in the sides and chest; medicine has little effect, and she seems to be fast approaching a state of consumption. From these symptoms I have oftener than once witnessed her rapid recovery, when it could only be attributed to a happy change in the affairs of her husband effecting in her a beneficial excitement.

In the above list, three of the patients, whose united ages amount to two hundred and fifty-one years, are females; the oldest of whom, a private patient, was attacked with rheumatism. The only parts affected with pain were the integuments of the head, and the muscles of the neck; there was little fever, and the symptoms soon yielded to small doses of pulv. antimonialis and pulv. ipecac. comp. taken morning and evening, with decoction of bark three times a day. A stiffness of the neck is still troublesome, the head projecting forward nearly in a horizontal position, position, and the patient is unable to raise it. Of the remaining Octagenarians, one is in comfortable circumstances, and throughout life has enjoyed good health; the equality, however, with which the functions have so long been performed is now broken, and the kidney is the first organ to give way; the urine is in small quantity, of a deep red colour, and great pain is felt in the region of the kidney, attended with fever, loss of appetite, and want of sleep; the bowels only perform their office when stimulated by strong cathartics, as calomel and cathartic extract in large doses: independent of the torpor incident to old age, this state of costiveness has probably been induced by the patient, from the first attack of her illness, having taken opium in considerable quantity. The medicines she has hitherto taken have not procured any relief; and as her strength is sinking, the probability is that she will not recover. The other patient is affected with cough and dyspnœa, and her chief regret is, that at 83 years of age she can no longer earn her daily bread.

SAMUEL FOTHERGILL.

*Southampton Street, Strand, June 21, 1806.*

